# Risk, Stigma, Trustworthiness, and Citizen Participation—A Multifaceted Analysis of Media Coverage of Dioxin Contamination in Midland, Michigan

**DOI:** 10.3390/ijerph16214165

**Published:** 2019-10-29

**Authors:** Jie Zhuang, Jeffrey G. Cox, Minwoong Chung, Joseph A. Hamm, Adam Zwickle, Brad L. Upham

**Affiliations:** 1 Department of Communication Studies, Texas Christian University, 2800 South University Drive, Fort Worth, TX 76109, USA; 2 Department of Communication Studies, Albion College, 611 East Porter Street, Albion, MI 49224, USA; jgcox@albion.edu; 3 Department of Communication, Michigan State University, 404 Wilson Road, East Lansing, MI 48824, USA; chungm12@msu.edu; 4 School of Criminal Justice, Michigan State University, 665 Auditorium Road, East Lansing, MI 48824, USA; jhamm@msu.edu (J.A.H.); zwicklea@msu.edu (A.Z.); 5 Environmental Science and Policy Program, Michigan State University, 293 Farm Lane, East Lansing, MI 48824, USA; 6 Department of Community Sustainability, Michigan State University, 480 Wilson Rd, East Lansing, MI 48824, USA; 7 Department of Pediatrics and Human Development, Michigan State University, 1355 Bogue Street, East Lansing, MI 48824, USA; upham@msu.edu

**Keywords:** agenda setting, dioxin contamination, health and environmental risk, environmental stigma, trustworthiness, citizen participation

## Abstract

In the United States, more than 200 communities are designated by the U.S. Environmental Protection Agency as areas of concern for dioxins. Informing the public about potential risks associated with dioxins and delivering information about how to avoid such risks are essential activities. News coverage of environmental and health problems affects how members of the public assess those problems in terms of both severity and how they are understood, as well as the extent of attention given to the problem by policy-makers. To contextualize public and institutional responses to dioxin contamination and remediation in a dioxin-affected community, we assessed 176 newspaper articles published over 30 years concerning dioxin contamination in Midland, Michigan, in terms of risk, trust in institutions, environmental stigma, and citizen participation. Articles about dioxin contamination and remediation in Midland appeared in both domestic and international newspapers. Domestically, both national and local newspapers covered this issue. The risks for human health and the environment caused by exposure to dioxins were widely covered, with much less media attention given to the trustworthiness of the organizations responsible for managing the risk, environmental stigma, and citizen participation. News coverage of these four themes also changed significantly overtime. Overall, our findings highlight the important role of local news media in communicating risk information, guiding safe behaviors, and facilitating community-level decision-making.

## 1. Introduction

Mass and specialty media are essential conduits for how members of the public come to know of, and learn about, environmental issues. The internet, world wide web, social media sites, television stations, newspapers, and radio interact in our information network community to orient viewers, readers, and listeners to social problems that can become appreciated as public issues by both members of the public and policy-makers [1]. The media influence public perceptions of events and issues cognitively, by conferring higher status to certain events and problems than to others [2], positively or negatively framing novel information [3], priming prior frames of reference to assist in the interpretation of new information [3], and through the common journalistic norms of balancing contrasting positions on issues [4,5]. The media also increasingly provide the public with the opportunity to voice their opinions and, sometimes, participate in decision-making processes [6]. In the context of environmental hazards, media portrayals of environmental issues shape the public’s environmental risk perceptions [7], influence perceptions of the actions taken by governmental and non-governmental agencies in coping with environmental hazards [8], and affect the public understanding of citizen participation in environmental controversies [9].

This study sought to assess how media coverage of an environmental hazard (i.e., dioxin contamination) changes over time and how events, new information, and the passage of time affected news media coverage in Midland, Michigan. We focused on four frames by which reporters, and thus subsequently readers, may have interpreted this long-running story: (1) environmental and health risks posed by dioxin contamination, (2) environmental stigma, (3) trustworthiness of involved organizations, and (4) citizen participation. The following sections will briefly review the agenda-setting theory, provide a contextual background for this investigation, propose research questions, and introduce our methodology and results.

### 1.1. Agenda-Setting Theory and Environmental Hazards

For over four decades, public agenda setting [10] has provided one of the most influential paradigms in media and communication research due to an extremely high correlation of issue importance in media coverage with issue importance among media consumers [11]. The agenda-setting theory was introduced to explain media’s capability in setting agendas and telling people what to think [10]. Subsequent research refined and extended this framework by conceptually and empirically developing it into a process of interactions among the media agenda, the public agenda, and the policy agenda [12,13].

In a review of media–risk research, four major roles played by media in setting the agenda about risks for the public are outlined [14], including (1) providing risk knowledge and informing citizens, (2) adjusting the public acceptability of risk, (3) motivating the public to take responsibility for and action in response to risk, and (4) providing imaginative schemata for voluntarily chosen risks. Framed within the agenda-setting theory, the present research aims to understand how newspapers fulfilled the first role of providing risk knowledge and informing the public by examining how the issue of dioxin contamination and remediation in Midland has been framed over time.

### 1.2. Context: Dioxins and Environmental Protection Agency (EPA) Areas of Concern

The chemical name “dioxin” refers to a family of hundreds of related chemical compounds, including 75 chlorinated dioxin congeners among which 2,3,7,8 tetrachlorodibenzo-p-dioxin (TCDD) is the most toxic [15]. Dioxin contamination of the environment has been a serious environmental problem in recent history. Notorious releases into the environment has been a consequence of the widespread use of dichlorodiphenyltrichloroethane (DDT) and agent orange, of which TCDD was a byproduct contaminant. Historically, concern for environmental pollution exploded with the contamination of Love Canal and Times Beach by many types of industrial chemicals which was considered to be “one of the largest environmental disasters in our nation’s history” [16]. Exposure to dioxins are developmentally toxic on the immune, liver and nervous systems, and can lead to a wide range of other adverse health outcomes, such as, gonadal and lymphoid atrophy, disruption of endocrine signaling, cardiovascular toxicity, bone, skin, and tooth toxicity, carcinogenesis, wasting, and lethality [17]. Residents in many of the most contaminated areas were evacuated, but concern remains, in terms of both new exposures and understanding how previous exposure to elevated levels of dioxin could continue to negatively influence people’s health [18].

In 1987, Michigan’s Tittabawassee-Saginaw-Bay Rivers watershed was designated as a federal area of concern (AOC) for dioxins. Dow Chemical Company’s industrial facilities in Midland, Michigan, are located on the banks of the Tittabawassee River and have been identified as a major source of dioxin pollution. This contamination was the result of incineration and water discharge over several decades [19], resulting in some of the highest known recorded levels of dioxin in the environment.

Multiple institutions have been involved in testing and remediation of the Tittabawassee River and its floodplain. This activity is ongoing and will continue downstream to the Saginaw River and then north to the Saginaw Bay of Lake Huron, altogether representing contamination that extends for more than 50 miles downstream from the Dow Chemical Company facility in Midland, U.S. In 2007, Dow Chemical was required by the EPA to start contamination clean up [20]. This process has involved close oversight by the Michigan Department of Environmental Quality (MDEQ). The Michigan Department of Human and Health Services (MDHHS) has also been active in the area through the development and dissemination of educational materials that provide advisories and recommendations for avoiding risky behaviors, such as eating locally caught fish.

Besides federal, state, and corporate stakeholders, community entities are also involved in the effort to reduce the harm caused by dioxins. In 2009, a Community Advisory Group (CAG) comprised of community volunteers was formed by the EPA. This group holds regular meetings to facilitate sustained discussions between the affected community and federal, state, and corporate stakeholders. In addition, a few local environmental groups, such as Lone Tree Council and Tittabawassee River Watch, remain actively engaged at a small scale.

Although efforts to understand pathways of dioxin exposures and to remediate the area through the removal of contaminated topsoil are underway, public perceptions of risk and individual-level responses to the environmental hazard have received less attention. Of the work that does exist, however, some suggest that people living in affected areas were shunned by new neighbors and that news media has intensified resident concerns [21]. We sought to better understand how media have contributed to communicating about this issue to the public by evaluating print media’s treatment of four themes: risk information, the trustworthiness of the organizations responsible for managing the risk, environmental stigma, and citizen participation.

### 1.3. Research Questions

The agenda-setting theory provides a perspective for understanding the role of media in society. This research had the primary objective of characterizing media coverage of one locality’s dioxin contamination and remediation experience. An important responsibility of media is to inform the public about health and environmental threats, so that people can make informed decisions to cope with the threats [14].

The literature of risk perception suggest that risk-related decisions and behavior are, in large part, determined by perceptions of two risk features, namely, vulnerability (i.e., how likely a threat exerts a direct and personal influence) and severity (i.e., how severe a threat is) [22], but factors such as uncertainty, controllability, and threat to future generations also play important roles. Thus, in addition to examining the extent to which news coverage reports that dioxin contamination is possible and causes serious consequences, this research also examined the degree of scientific uncertainty regarding the effects of dioxins on humans and the environment for two reasons. First, previous research indicates that when uncertainty in the scientific community is picked up by the public through media’s presentations of risks, the public’s willingness to partake in policy-making and take actions in support of policy tends to decrease [9]. Second, news media are responsible for reporting issues in a balanced and unbiased manner, and one way to do so is by highlighting the uncertainties surrounding science and scientific findings [23,24]. However, in the case of dioxin contamination, the representation of uncertainties of the scientific findings can be misleading. This study also investigates the extent to which pathways to dioxin risks (e.g., consumption of local produce) are discussed in news articles. The first research question (RQ) addresses these concerns:

**RQ1**: How has dioxin contamination in Midland, Michigan, been described in newspaper articles, especially in terms of environmental and human health risks and pathways of exposure?

Environmental contamination is a primary driver of environmental stigma [25]. Environmental stigma is defined as “an inherent property of any discovered or anticipated change to a community, an object, a product or a place due to its association with exposure to a toxic substance” [26] (p. 21). Contaminated properties are considered dangerous, even if they are later remediated; owners of contaminated properties also tend to be vulnerable to social distancing and financial loss as a result of market devaluation of their properties. The decrease in market values of contaminated properties has been used as a direct measure for environmental stigma [27,28], and research shows properties in close proximity to a hazardous site also suffer from negative economic effects [29]. Areas contaminated by chemical toxins are stigmatized and shunned by anxious publics that perceive dreadful health and environmental effects [30,31]. Local residents in Midland and Saginaw, Michigan, do feel stigmatized for living in a contaminated area [21]. However, research about whether and to what extent newspaper coverage may add to or reinforce this sense of stigmatization is slight. We asked:

**RQ2**: To what extent was the dioxin contaminated area in Midland, Michigan, and the residents living in the area portrayed negatively in newspaper coverage?

Media stigmatization of environmentally hazardous places may be related to distrust of responsible parties [32]. The extent to which responsible parties such as Dow Chemical Company are portrayed as trustworthy may play an important role in shaping public perceptions about and reactions to environmental policies [33]. Reliance on information delivered by responsible parties influences the extent to which citizens perceive environmental risks and engage in public forums and decision-making bodies related to the contamination [34]. In the specific context of this study, multiple agents, such as U.S. EPA, MDEQ, MDHHS, and Dow Chemical Company, participate in educating residents in the affected communities about the potential health and environmental harms caused by dioxin contamination and the remediation process. These organizations have hosted public meetings and engaged in outreach to individuals (e.g., mailing educational materials to local residents). Research suggests that residents’ trust in these organizations is associated with risk-relevant behavior [35], but what about media coverage? Thus, we asked:

**RQ3**: How has the trustworthiness of the organizations involved in dioxin contamination and remediation been portrayed in newspaper articles?

Citizen activation and participation are significant parameters influencing the formation and implementation of environmental policies [36]. Successful and effective citizen participation not only supports good environmental decision-making but also has the potential to build community and fertilize constructive community relationships [37]. In the arena of political participation, research shows that exposure to media political content has a positive impact on public political participation [38]. Researchers have found that lack of public awareness of environmental problems leads to decreased citizen activation and participation in policy formation and subsequently to non-compliance of citizens with environmental policies [39]. Traditional media, especially local TV stations and newspapers, have the potential to play critical roles in informing the public about the opportunities for participation in decision-making. Throughout the course of dioxin contamination and remediation in Midland, multiple public meetings were held, public comments sought along, with several educational efforts by mailing educational materials. Nevertheless, this community has been characterized as “disengaged” on this issue [21]. Little is known regarding whether and how news media have disseminated information about the opportunities that citizens can take part in policy-making and have their voice heard:

**RQ4**: How was citizen participation in policy-making concerning dioxin contamination and remediation in Midland, Michigan, described in newspaper articles?

How media cover important issues changes over time. The agenda-setting hypothesis states that public opinion is reflective of the extent and prominence of media coverage. Prior research shows that changes in media coverage of climate change exerted a significant impact on public concerns about climate change [40]. Similarly, coverage of AIDS progressed through several stages over time [41]. Dioxin contamination in Midland has had a long history, throughout which media have paid ongoing attention. However, little is known regarding whether and how the themes and content regarding dioxin contamination have shifted. Therefore, we asked:

**RQ5:** How does news article coverage of (1) risk, (2) environmental stigma, (3) trustworthiness of involved organizations, and (4) opportunities for citizen participation change over time?

Research about environmental journalism and communication has shown that newspapers vary in the ways they report environmental issues [1,42]. Multiple factors drive variation, ranging from news media status in social power hierarchy [43] to the manipulative power of public relations by news sources [44], to individual journalists’ political orientation and subjective beliefs [11,44]. It was found that for global warming, national news agents, especially The New York Times and a few other national news organizations, served as the initial information sources that raised public awareness and provided information [42]. However, in the context of regional environmental pollution, it is less clear whether and how national and local newspapers might differ in how they report such issues:

**RQ6:** Do local and national newspapers differ in their coverage of dioxin contamination and remediation in Midland Michigan?

## 2. Method

### 2.1. Sampling

Two datasets, LexisNexis Academic and NewsBank, were used to search for newspaper articles with keywords “dioxin contamination”, “Midland Michigan” and “EPA area of concern”. From 1979 to 15 June 2016, the search produced 181 total newspaper articles. We excluded law appeals, court decision letters, and articles that were written in languages other than English, producing a final set of 176 articles for analysis.

### 2.2. Codebook Development

The codebook consisted of two sections: Basic information of news coverage (e.g., year of publication, length, type of coverage, major theme, secondary theme), and focal features of news coverage (e.g., risks related to human health, environmental stigma, trustworthiness of involved agencies, and citizen participation).

#### 2.2.1. Basic Information

Basic information about the news coverage we collected included article length (measured in the number of words), major theme (dioxin contamination, dioxin remediation, EPA regulation, community opinions, local government opinions, community advisory group meetings, and other), and secondary themes (secondary themes had the same coding categories as the major theme). Each article was coded to have only one major them but could have multiple secondary themes.

#### 2.2.2. Focal Features of News Coverage

**Risks.** We coded the news articles on the basis of whether risks to humans (e.g., potential for causing various diseases including cancer) and the environment (e.g., adverse effects on vertebrate spices) were described, whether the severity of such risks was described, whether the risks were described as definitely negative, uncertain/controversial, or no effect, whether the article noted that human beings are susceptible to dioxins, and whether dioxin exposure pathways were addressed (via natural environment, consumption of fish, chicken/eggs, dairy products, or other food).

**Stigma.** Stigma was examined in the news articles from three dimensions: Reporting of market values of affected properties, comparison between Midland and nearby communities and other EPA areas concerning the environment, and reporting of how residents in other communities stigmatize dioxin contamination in Midland.

**Trustworthiness of involved organizations.** Articles were also coded for the three dimensions of trustworthiness [45]. Ability was defined as the belief that a trustee has the power to do what needs to be done, benevolence was defined as the extent to which a trustee is believed to desire to do good for a trustor, and integrity was conceptualized as the belief that a trustee makes good-faith agreements, tells the truth, acts ethnically, and fulfills promises. These dimensions were measured as directed at four of the organizations involved in this case of dioxin contamination and remediation (U.S. EPA, MDEQ, MDNR, and Dow Chemical Company).

**Citizen participation.** Opportunities for participation was operationalized as: (1) Whether reactions to dioxin remediation from local environmental interest groups, general public, and the community advisory group were mentioned in the news article, and (2) The valence of their responses (i.e., positive and supportive, mixed, negative, challenging, and concerned).

### 2.3. Training, Intercoder Reliability, and Coding

A 4 h training was conducted by an author and a graduate student coder who was blinded to the research questions. The coder coded 10 news articles that were randomly selected from the dataset to familiarize the coder with the codebook. Ambiguity was addressed through discussion. Afterward, the author and the coder coded 25 news articles randomly selected from the data and compared coding to assess inter-coder reliability. Krippendorff’s alphas were calculated for each category, and reliability for each category was 0.79 or higher. After discussing and solving the discrepancies, the coder proceeded to code the remaining articles.

## 3. Results

### 3.1. Preliminary Results

The length of the articles varied considerably, with an average length of 981 words (median 786.4 words, *SD* = 1392.17 words, ranging from 19 words to 15,479 words). In total, 51.7% of the articles (n = 91) were published in national newspapers, such as The New York Times and the Washington Post, 22.2% (*n* = 39) were published in local newspapers including the Midland Daily, Bay City Times, and Saginaw News, 6.3% (*n* = 11) were published in newspapers in other municipalities, such as the cities of Grand Rapids and Traverse City, and 19.9% of all the articles (*n* = 35) were published in foreign newspapers.

In terms of major themes, 34.7% of the news articles (*n* = 61) focused on discussion of dioxin contamination, followed by remediation and cleanup effort (21.0%, *n* = 37); 3.4% of the articles (*n* = 6) discussed EPA regulation, 2.3% (*n* = 4) reported community advisory group meetings, and 1.7% (*n* = 3) presented community opinions about dioxin contamination and/or remediation. The major theme for 36.4% of the articles (*n* = 64) were coded as “other”, including topics such as court appeals, lawsuit announcement, and advertisements of the Dow Chemical Company.

### 3.2. Research Questions

**RQ1**: Coverage about Risk and Pathways to Risk

News coverage reported risks to both humans and the ecological environment. Over half of the news articles (54%, *n* = 95) reported dioxin-related human health risk, including short-term effects such as skin lesions and long-term effects such as impairment of the immune system. For example, one article mentioned nearby residents’ fear that dioxin in the soil and water would cause soft-tissue sarcoma and birth defect, and both were found in higher than normal levels in the Midland area. On the other hand, how dioxins might affect human health was downplayed in some articles. Another article mentioned extremely high levels of dioxins were only found in two isolated locations and “there was no threat to the health of Midland residents”. Forty-six percent of the articles (*n* = 81) reported dioxin-related risk to the environment, such as harms to vertebrate spices and air pollution. For instance, one article mentioned that some Michigan environmental groups “are increasing their demands for official action against dioxin poisoning in light of what they regard as pervasive evidence of pollution by chlorinated dioxins”.

Among articles that mentioned dioxin-related human health risk, 56.8% (*n* = 54) reported that dioxins had definitely negative health effects (e.g., an article mentioned that dioxins in residential soil may present an “imminent and substantial endangerment of the health of those living in the area, especially children”), whereas 22.2% (*n* = 39) stated that the effect of dioxin on human health was uncertain (e.g., an article stated no plan was made to determine “what results of exposure are”). In terms of environmental risk, 62.9% in which environmental risk was mentioned (*n* = 51) stated that dioxins would affect the environment negatively (e.g., soil and water were poisoned; dioxins penetrated the soil and remained there infinitely), whereas 32.1% (*n* = 26) stated that the effects of dioxins on the environment were uncertain (e.g., animals appeared unlikely affected by dioxins).

In terms of susceptibility, 48.3% of the entire sample (*n* = 85) portrayed humans as susceptible to the effect of dioxins. Regarding the exposure pathways, 38.6% (*n* = 68) mentioned that humans could be exposed to dioxins via the natural environment (e.g., dioxins are “widespread in nature and may be a natural byproduct of combustion from incinerators”), and 22.2% (*n* = 39) mentioned that consumption of fish would cause humans to be exposed to dioxins (e.g., reports advocated “a ban on eating fish from the bay”). News articles that mentioned other pathways to dioxins, such as consumption of locally raised chickens/eggs and locally produced dairy products or other foods were rare, ranging from zero to 5.1%.

**RQ2:** Environmental Stigma

Environmental stigma was not a major theme. In fact, 2.8% of the articles (*n* = 5) mentioned decline in affected properties’ market values, 1.7% (*n* = 3) negatively compared Midland with other communities, and 5.7% (*n* = 10) mentioned local residents’ negative feelings about their community being contaminated (e.g., some Midlanders complained that their town was being unfairly depicted as “an environmental disaster area”).

**RQ3**: Trustworthiness of Involved Organizations

Trustworthiness of involved agencies was coded for (1) federal government, (2) state government, (3) EPA, and (4) Dow Chemical Company, on general trust, ability, benevolence, and integrity. Although EPA is a federal agency, it was separated from the analysis, given its significant role in dioxin contamination and remediation. For federal and state governments, the news articles minimally mentioned their levels of trustworthiness, ability, benevolence, and integrity. When federal or state agencies were mentioned, only one article portrayed them with a lack of trustworthiness. For EPA, 3.4% of the articles mentioned a lack of trustworthiness. Approximately 5% of the articles mentioned lack of integrity in relation to the EPA.

Eight articles portrayed Dow as trustworthy (e.g., an interviewee stated that “he believed Dow’s effort, to be completed within two years, will help answer concerns about the perils of dioxin”. In another article, an interviewee said “I am not a scientist but I have faith in Dow’s experts”). Twenty articles portrayed the company negatively (e.g., in discussion of an internal study conducted by Dow which showed low mortality rates associated with elevated dioxin levels, one article mentioned a criticism by stating “XXX discounted the study on the ground that it had not been made public and subjected to outside scrutiny”). Five articles portrayed Dow Chemical as lacking benevolence (e.g., an article quoting a mother’s opinion: “we want an independent assessment from Dow of this area to find out the truth about dioxin”). Seventeen articles doubted the company’s integrity (e.g., in discussing the validity of data and information from Dow, one article mentioned “it is unclear whether the data could be obtained from Dow … the agency is suing the company for refusing to disclose”), with nine articles affirming its integrity.

**RQ4**: Citizen Participation

Citizen participation was coded for (1) presence of environment interest groups, individual residents, community advisory group, and local government, and (2) the valence of their responses to dioxin cleanup efforts. We found that 14.8% of the articles (*n* = 26) mentioned environment interest groups, and in the vast majority of these articles (*n* = 24), environment interest groups were portrayed as responding negatively or challenging cleanup efforts (e.g., an article mentioned, quoting a member of a local environmental group: “we need our government to issue a clear scientific statement and report on the toxicity of this chemical … it appears it’s probably politics as usual”). In 11 articles, individual residents’ responses were mentioned, with 8 articles portraying them as negative and 3 with mixed evaluations. Eight articles mentioned local government’s responses, with three of them negative (e.g., an article mentioning local government along with other agencies “are calling on the U.S. Environmental Protection Agency to reconsider its stricter interim preliminary remediation goals for dioxin”), one positive, one mixed, and three other articles with no clear valence mentioned. The response from community advisory groups was only mentioned in one article and was portrayed negatively.

**RQ5**: Over-Time Change

**Figure 1:**
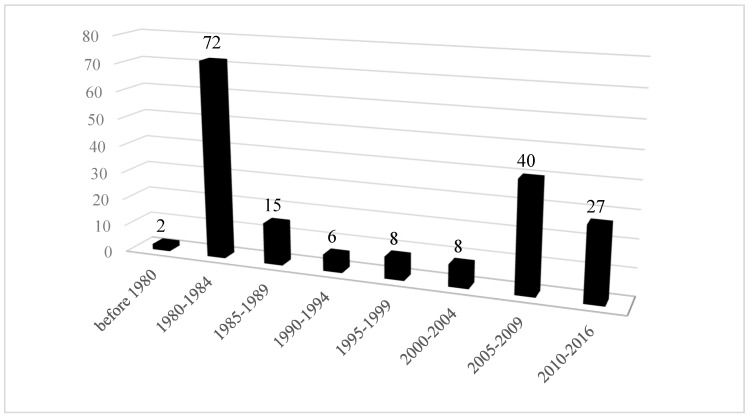
Amount of news coverage over time.

**Figure 2:**
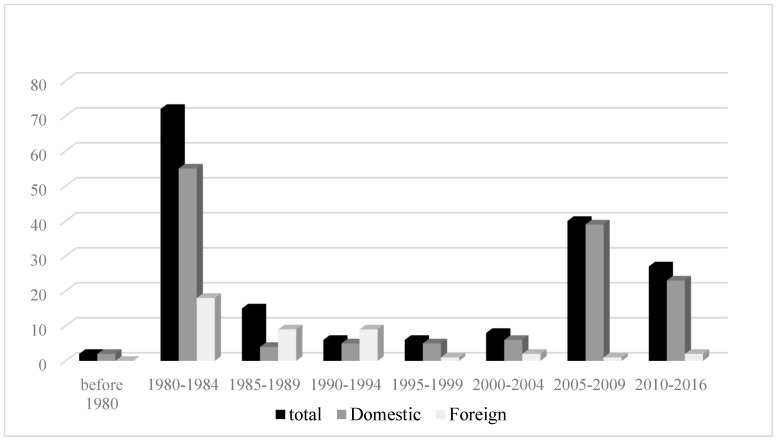
Over-time amount of news coverage by native and foreign newspapers.

**Figure 3:**
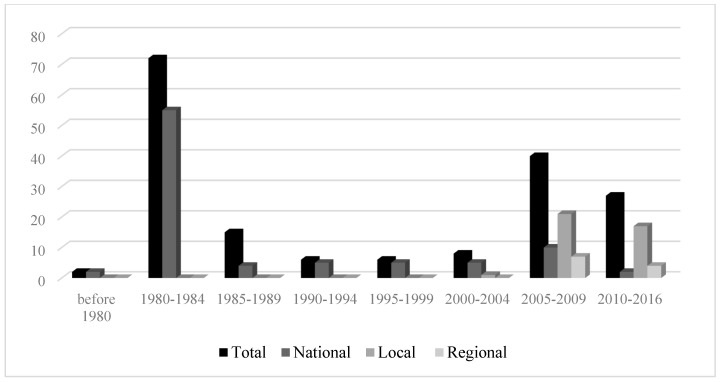
Over-time amount of news coverage in national, regional, and local newspapers.

To examine the over-time changes in these variables, years of publication for the news articles were collapsed into five-year ranges, (pre-1980, 1980–1984, 1985–1989, 1990–1994, 1995–1999, 2000–2004, 2005–2009, 2010 and after). The results showed a bi-modal distribution. The amount of news coverage peaked in the 1980–1984 timeframe and dropped afterword until 2004. In the 2005–2009 time period, the amount of news coverage increased (Figure 1) and then showed a slight decline from 2010 onward (see Figure 1). There were more news articles published in U.S. newspapers. It was clear that prior to 2005, dioxin contamination and remediation in Midland was mainly covered in major domestic newspapers and foreign newspapers such as the Globe and Mail (Canada) (Figure 2). After 2005, local and regional papers published more articles (Figure 3).

**Figure 4:**
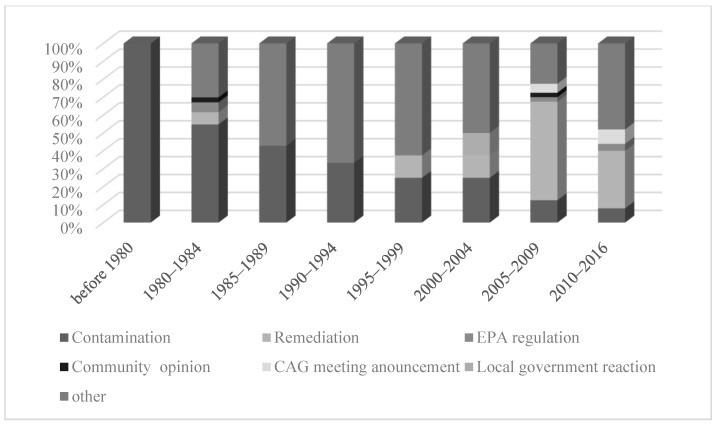
Over-time change in major themes in news coverage.

The themes changed over the years. Before 1994, contamination was the major and dominant theme for news articles (except during 1980–1984). Since 1995, the theme of remediation became salient, especially during 2005–2009 and 2010–2016 (Figure 4).

**Figure 5:**
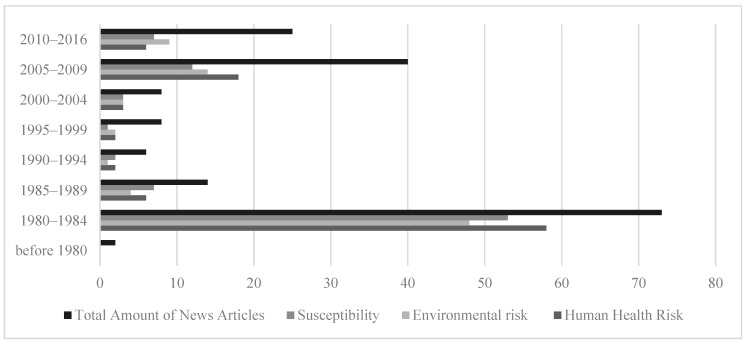
Over-time news coverage of human health and environmental risk of dioxin as well as susceptibility linked to dioxin contamination.

In terms of changes in the coverage of dioxin-related risk, the results showed that prior to 2010, newspapers focused on reporting human health risk more than environmental risk. After 2010, journalistic attention to environmental risk exceeded the attention to human health risk. The coverage about human susceptibility also changed over time, such that susceptibility was reported heavily during 1980–1984, then dropped until 2005–2009 (Figure 5). In general, the graphic results show a bi-modal distribution for reporting of human health and environmental risk and human susceptibility.

**RQ6**: Differences between National and Local Newspaper Coverage

To examine the difference between national and local newspaper coverage, we conducted chi-square analyses. First, we found a significant difference between national and local newspapers for the major themes covered, χ² (14) = 72.86, *p* < 0.001. National newspapers had more coverage of dioxin contamination than did local newspapers. Local newspapers focused more on dioxin remediation and cleanup efforts.

National newspapers also differed from local newspapers with significantly more coverage of human health risk, χ² (2) = 24.16, *p* < 0.001, environmental risk, χ² (2) = 14.96, *p* < 0.01, and humans’ susceptibility to dioxin contamination, χ² (2) = 31.25, *p* < 0.001. The articles coded for institutional trust, environmental stigma, and citizen participation were too few to conduct chi-square analyses.

## 4. Discussion

News media have been historically blamed for misinforming and misleading the public regarding the danger of various risks, such as nuclear power, radiation, and cancer [46,47]. This can be attributed to communicators’ own limitations in interpreting scientific findings and the inherent complexity of risk assessment. To effectively inform and educate the public about risk issues, communicators must recognize and overcome their own limitations in “scientific risk assessment” [22] (p. 403).

News organizations have given extensive coverage to environmental hazards over the years [1,25,48]. How news media report environmental hazards can impact public risk perceptions, as news media serve as a primary information source about risks and hazards [49,50]. Our study focused on dioxin contamination in the company town of Midland, Michigan [51]. We conducted a content analysis to understand how newspaper coverage presented this issue concerning human health and environmental risk, trustworthiness of involved organizations, environmental stigma, and citizen participation. We coded 176 newspaper articles published over a 30-year period. Despite its dreadful impact on human beings and the natural environment, dioxin contamination does not appear to attract much scholarly attention in social science. This study, to our best knowledge, is one among few others [52] which analyzed newspaper coverage of a specific environmental pollutant at a location that has a long history of dioxin contamination and is currently undergoing remediation.

The data revealed a bi-model distribution for major themes covered in the newspaper articles, with contamination and remediation receiving the most newspaper attention. Regarding the four content topics of risks, environmental stigma, institutional trust, and citizen participation, the coverage of risks predominated. Approximately half of the newspaper articles reported dioxin-related human health or environmental risks. Although these risks were largely portrayed as negative, the uncertainties regarding the effect of dioxin on human and environmental health were mentioned in many articles. Previously, researchers have pointed out that news coverage tends to translate scientific uncertainty into certainty [53]. Researchers argue that journalists portray scientific discourses as uncertain by providing different perspectives on the same scientific issue in order to achieve the goal of balance, with a result of increased public perception of scientists’ credibility [9,36].

Future research could examine how the (un) certainty of risks caused by dioxin contamination as represented in newspaper articles affects public perceptions regarding dioxin contamination and public active participation in risk-management decisions and policy-making. In comparison to the coverage of risks, the newspaper articles we studied paid less attention to residents’ susceptibility to risks. Fewer than half of the newspaper articles studied stated that human beings were susceptible to the effects of dioxin, and when mentioning the extent to which humans are susceptible, fewer than 40% of the articles discussed exposure pathways, indicating primarily the natural environment and consumption of locally caught fish. Exposure to other foods which contribute more than 90% of human exposure to dioxins [54] was rarely mentioned. Future research is desired to test the direct relationship between limited media coverage of pathways to dioxins and local residents’ consumption of locally produced food, such as dairy products.

Environmental stigma and trustworthiness of involved organizations were not much represented. Only small percentages of newspaper articles mentioned a decline in property values and residents’ negative feelings about their communities being contaminated. Dow Chemical Company was mentioned more often than government agencies, with most of those mentions being negative rather than positive. Similarly, citizen participation received very few mentions. Most of the reported community responses to dioxin cleanup efforts were negative and critical. The minimal coverage of public participation could be attributed to low levels of engagement, the media’s overlooking of such engagement, and, possibly, the lack of interest of the general public in this issue. Indeed, previous research has shown a lack of community engagement, as this issue was not ranked as a priority issue in this same community [21]. A direct connection was found between a diminished news environment and a decrease in citizen engagement during the 2010 U.S. House election, which speaks directly to the role of news media in shaping civic participation [55]. An increasing corpus of research has shown the powerful influence of exposure and contribution to social media on citizen participation in policies [55]. Future research is desired to examine how exposure to various media (i.e., national, local, and social) influences citizen participation in policy-making and further inform the policy agenda.

We also found changes in the amount of newspaper coverage given to this issue. Specifically, the results showed that when dioxin contamination in Midland was first discovered in the early 1980s, the issue generated considerable coverage. Media attention soon diminished, followed by a second peak of interest when residents and government officials in the affected area began discussing cleanup. Findings also show transitions in media themes. Early on, articles focused on reporting the environmental and human risks associated with dioxin contamination, which lost media attention later. Discussion about EPA regulation became more salient in newspaper coverage over the time of investigation. Our findings also demonstrate that dioxin contamination and remediation received attention from newspapers at different levels, initially with coverage by national and foreign newspapers and later in local media. National coverage was attuned to the effect on share prices of Dow Chemical Company as a publicly traded firm.

## 5. Conclusions

Although this research strives to provide a comprehensive account of newspaper coverage of a long-running environmental contamination issue, this study has limitations. First, we only analyzed content in newspapers. Different types of media may have covered this issue differently [56,57]. In future research, special attention should be given to how discussion surrounding dioxin contamination and remediation unfolds on social media, how the information presented on social media regarding this issue is similar or different from what is available in traditional media, and what differential effects traditional and new media make on the public and policy agenda [15]. Second, the data were only descriptive. The agenda-setting theory has been used to explain how media coverage influences public cognition as well as policy decisions, including recursive effects [2,6,16]. Our study was limited to an assessment of media coverage. Third, by choosing one site, though arguably the most prominent dioxin site in the U.S. during the time period of study, we are unable to generalize to other EPA areas of concern for dioxin.

Despite these limitations, this study adds to the literature by providing a comprehensive account of media coverage of dioxin contamination in a community where dioxin contamination has a long history. It can be argued that, while content analysis of the coverage about climate change [58,59] is important, toxic site-specific contamination poses more immediate and severe threats to human health and represents a type of risk that is likely more attractive to news professionals for this reason. This study highlights the import role of news media, especially local news media, in informing the public about the health risks associated with dioxin. This information not only guides the public towards safer behaviors considering contamination, but also provides opportunities for local residents to express their voices and partake in community level decision-making. Moreover, as argued in [54], one obstacle that hampers our understanding about the connection between news media and civic engagement is the lack of systematic analyses of the media content. This research helps set the stage for future research linking media exposure and civic engagement in environment-related policies.
